# Exploring the Potential of Gold Nanoparticles in Proton Therapy: Mechanisms, Advances, and Clinical Horizons

**DOI:** 10.3390/pharmaceutics17020176

**Published:** 2025-01-30

**Authors:** Giorgio Giuseppe Carbone, Stefania Mariano, Alessandra Gabriele, Sabrina Cennamo, Vitantonio Primiceri, Muhammad Rizwan Aziz, Elisa Panzarini, Lucio Calcagnile

**Affiliations:** 1 CEDAD (Center of Applied Physics, Datation and Diagnostics), Department of Mathematics and Physics “E. De Giorgi”, University of Salento, 72100 Lecce, Italy; giorgiogiuseppe.carbone@unisalento.it (G.G.C.); alessandra.gabriele@unisalento.it (A.G.); sabrina.cennamo1@gmail.com (S.C.); vitantonio.primiceri@unisalento.it (V.P.); muhammadrizwan.aziz@unisalento.it (M.R.A.); lucio.calcagnile@unisalento.it (L.C.); 2 Department of Mathematics and Physics “E. De Giorgi”, University of Salento, 72100 Lecce, Italy; 3 Department of Biological and Environmental Sciences and Technologies, University of Salento, 73100 Lecce, Italy; elisa.panzarini@unisalento.it

**Keywords:** gold nanoparticles, proton therapy, radiosensitization, cancer therapy, reactive oxygen species, precision medicine

## Abstract

Proton therapy represents a groundbreaking advancement in cancer radiotherapy, leveraging the unique spatial energy distribution of protons to deliver precise, high-dose radiation to tumors while sparing surrounding healthy tissues. Despite its clinical success, proton therapy faces challenges in optimizing its therapeutic precision and efficacy. Recent research has highlighted the potential of gold nanoparticles to enhance proton therapy outcomes. Due to their high atomic number and favorable biological properties, gold nanoparticles act as radiosensitizers by amplifying the generation of secondary electrons and reactive oxygen species upon proton irradiation. This enhances DNA damage in tumor cells while preserving healthy tissues. Additionally, functionalization of gold nanoparticles with tumor-targeting ligands offers improved precision, making proton therapy more effective against a broader range of cancers. This review synthesizes current knowledge on the mechanisms of gold nanoparticle radiosensitization, preclinical evidence, and the technological hurdles that must be addressed to integrate this promising approach into clinical practice, aiming to advance the efficacy and accessibility of proton therapy in cancer therapy.

## 1. Introduction

Proton therapy (PT) represents one of the most sophisticated and promising approaches in cancer radiotherapy. By utilizing high-energy proton beams, PT selectively targets cancer cells, damaging their DNA while preserving surrounding healthy tissue [1,2,3]. The key advantage of PT lies in its highly precise spatial energy distribution, characterized by the Bragg peak, which ensures that the maximum radiation dose is delivered at the tumor site, minimizing exposure to adjacent tissues [4]. This feature improves therapeutic outcomes while reducing side effects, thus enhancing patient quality of life [5]. The mechanism occurs because protons lose energy progressively as they travel through the body, with a sharp rise in energy deposition at the end of their path, known as the Bragg peak [6]. In clinical practice, proton energies typically range between 70 MeV and 250 MeV, depending on the depth of the tumor. Lower energy protons (70–100 MeV) are used for superficial tumors, while higher energies (200–250 MeV) are required for deeper tumors [7]. The treatment is often delivered via passive scattering, a technique that uses scatterers and bolus materials to broaden and shape the proton beam to match the tumor’s size and shape [8]. Although less precise than pencil beam scanning, passive scattering can be useful for larger or irregularly shaped tumors. Proton therapy typically delivers doses in the range of 1.8–2.2 Gy per fraction, similar to conventional photon therapy [9]. Total doses for a treatment course usually range from 60 to 80 Gy, depending on the tumor type and treatment protocol. Proton therapy has shown particular benefits in pediatric patients and tumors near critical structures, where minimizing damage to healthy tissue is paramount [10]. These characteristics make proton therapy a promising and effective option for certain cancer treatments.

Initially conceptualized by Wilson in 1946, PT has seen significant development since its first clinical application in the 1950s [11]. Despite its growing clinical adoption, challenges remain, particularly in optimizing beam scanning techniques and understanding the distinct biological effects of proton radiation compared to photons [12].

Technological advancements in radiotherapy oncology have progressively led to more refined and personalized treatment options, significantly improving patient outcomes. One of the core principles guiding modern radiotherapy, including PT, is the ‘as low as reasonably achievable’ (ALARA) principle. This principle emphasizes the importance of delivering an optimal therapeutic dose to the tumor while minimizing radiation exposure to surrounding healthy tissues [13].

The primary objective remains maximizing tumor control while reducing the risk of radiation-induced toxicity in normal tissues [14]. PT, while highly effective in treating deep-seated, inoperable, or recurrent tumors [2,3], still faces several technological challenges. These challenges revolve around further improving the precision of proton beam localization and enhancing its overall therapeutic effectiveness.

One of its key advantages lies in the Bragg peak, a phenomenon where protons deposit most of their energy at a specific depth in tissue, just before they stop.

Current efforts are focused on developing strategies that can optimize the spatial and biological targeting of the proton beam. One promising direction involves exploring novel sensitizing agents, such as nanoparticles, which have the potential to amplify the effects of PT [15]. These agents can increase the therapeutic impact on cancer cells by enhancing the radiation dose delivered to the tumor without increasing damage to adjacent healthy tissues. The integration of such sensitizing agents into PT holds the potential to expand its applications, offering more effective treatment options for a broader range of cancers [16].

However, despite significant progress, PT still requires ongoing research to refine these approaches and achieve more consistent, targeted therapeutic outcomes. Technological innovations aimed at improving the precision and efficacy of PT remain crucial for its future development. Further studies are essential to overcome the existing limitations and translate these advancements into routine clinical practice, thereby improving patient care and expanding the therapeutic scope of PT [17].

To address these challenges and enhance the efficacy of PT, researchers have explored novel strategies, including the incorporation of gold nanoparticles (AuNPs).

AuNPs have garnered attention for their potential to enhance PT. Due to their high atomic number and favorable biological properties, AuNPs can significantly amplify radiation effects. When irradiated with protons, AuNPs increase the generation of secondary electrons and reactive oxygen species (ROS), leading to enhanced DNA damage in tumor cells. This radiosensitization effect has been shown to increase the efficacy of PT while maintaining the precision required to limit damage to healthy tissues. AuNPs can also be functionalized with specific ligands to improve tumor targeting, further enhancing treatment accuracy. Preclinical studies have demonstrated that AuNPs, when combined with PT, not only enhance DNA damage but also delay tumor growth, suggesting that this approach could lead to more effective cancer treatments with fewer side effects [18].

Given the potential of AuNPs to significantly improve PT outcomes, this review aims to synthesize current knowledge and provide an overview of recent advances in this field. In the following sections, we will explore the underlying mechanisms of AuNP radiosensitization, recent preclinical findings, and the challenges that need to be addressed to translate this technology into clinical practice.

## 2. Role of Nanoparticles in Cancer Therapy

In recent years, the application of metallic NPs in PT has attracted substantial research interest due to their potential to enhance radiation dose delivery selectively to cancer cells. Metallic NPs, especially those made from high-atomic-number (Z) elements, are advantageous for radiation therapy, as their atomic structure facilitates greater absorption of energy from proton beams, thereby intensifying DNA damage in targeted tumor cells [19]. For instance, Sisin et al. demonstrated that the use of metal-based NPs as dose enhancers leads to significant improvement in the therapeutic efficacy of PT in vitro [20]. Additionally, a study by Kim et al. found that animals injected with metal nanoparticles before undergoing PT achieved a remission rate of 33–65%, as opposed to a 25% remission rate in animals treated with protons alone, underscoring the enhanced radiosensitization provided by metallic NPs [21].

**Figure 1:**
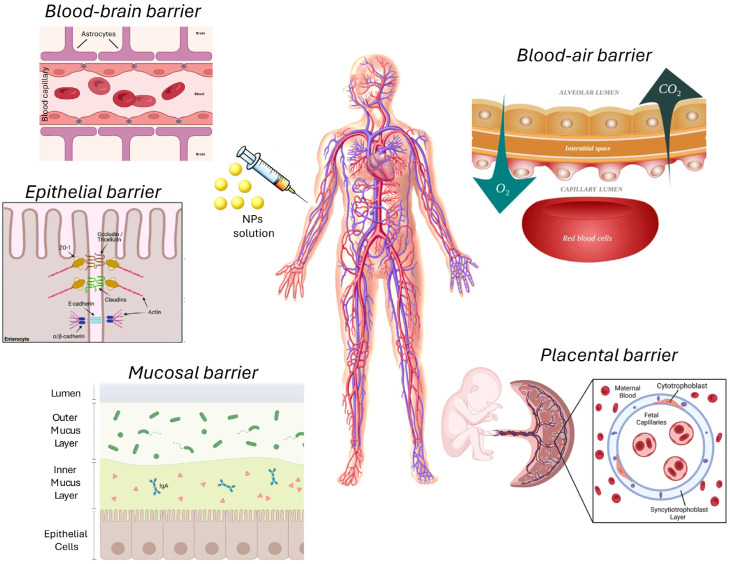
Main biological barriers that NPs must overcome to enable precise drug delivery. More advanced NP designs, which enhance delivery efficiency, can substantially increase the effectiveness of precision medicines, thereby speeding up their transition to clinical use.

Despite the advantages, the efficacy of NP delivery is often hampered by biological barriers such as the tumor microenvironment (TME) and blood–brain barrier (BBB) (Figure 1). Overcoming these barriers is essential for efficient NP-based therapies, and strategies such as PEGylation or active targeting with antibodies are being studied to improve nanoparticle accumulation in tumor tissues [22].

Among the various metallic NPs explored, those containing elements such as gold, silver, and iron oxide are highly studied due to their unique physical properties, biocompatibility, and stability under physiological conditions [23].

However, nanoparticles such as platinum nanoparticles (PtNPs), titanium dioxide nanoparticles (TiO₂ NPs), silver nanoparticles (AgNPs), and gadolinium nanoparticles (GdNPs), have also demonstrated radiosensitization capabilities, although with distinct advantages and limitations. Table 1 shows some examples:

AuNPs stand out for their remarkable attributes, making them particularly promising in PT. AuNPs exhibit several notable advantages over other metal-based nanoparticles. Due to gold’s high atomic number (Z = 79), AuNPs achieve greater energy absorption from proton beams, which can result in enhanced localized energy deposition and consequent cell damage in tumors [30]. Furthermore, AuNPs are biocompatible and have customizable surfaces, allowing for functionalization with targeting molecules like peptides or antibodies, thus increasing the specificity of delivery to cancerous cells [31]. AuNPs’ tunable sizes and shapes also allow them to be optimized for enhanced permeability and retention (EPR) in tumors, providing additional benefits in terms of selective accumulation in cancerous tissue [32]. These attributes collectively highlight the unique position of AuNPs in advancing therapeutic strategies.

Moreover, AuNPs also present certain challenges that must be addressed to fully exploit their potential in PT. One limitation is their tendency to accumulate in non-targeted organs, which can lead to toxicity. Moreover, despite their biocompatibility, there are concerns about the long-term persistence of gold within the body, as it is not readily metabolized. Consequently, future research should focus on enhancing the biocompatibility of AuNPs while reducing off-target effects. For example, investigations into biodegradable coatings or hybrid nanoparticles that combine gold with more metabolizable materials could provide solutions to these issues. Additionally, more studies are needed to optimize the concentration and size of AuNPs for maximal radiosensitizing effects, minimizing the risk of toxicity.

Further research is also essential to clarify the mechanisms by which AuNPs enhance PT efficacy, including the generation of reactive oxygen species (ROS) and other secondary particles that may amplify DNA damage in cancer cells. Understanding these mechanisms could lead to the development of even more effective nanoparticle-based therapies, potentially incorporating multimodal strategies that integrate imaging and treatment within a single nanoparticle platform. Combining real-time imaging techniques (like MRI, PET, or CT scans) with nanoparticles to guide the delivery of treatment more precisely. Nanoparticles can be modified to respond to specific imaging signals, allowing clinicians to track the particles as they accumulate in the tumor [33]. Such strategies are crucial for guiding radiation beams with precision, as they provide enhanced tumor visualization and real-time monitoring [34]. This can help optimize treatment planning by ensuring that the therapeutic agents are accurately targeted, improving efficacy and minimizing side effects [35].

## 3. Methods of Synthesis of AuNPs

**Figure 2:**
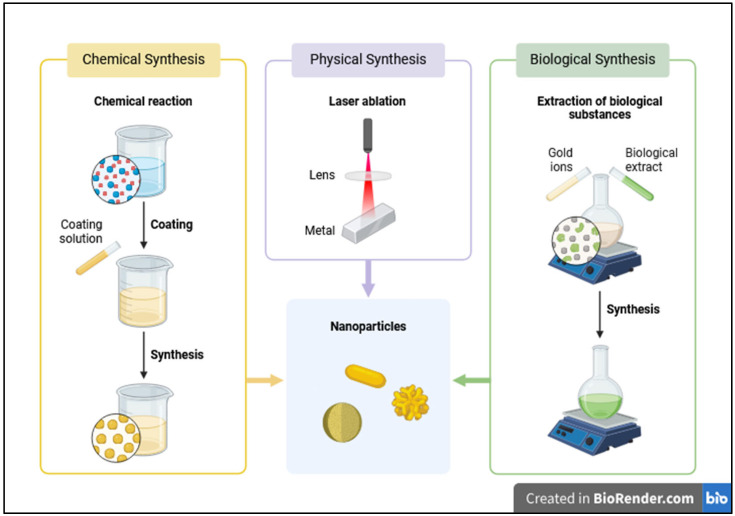
Scheme of different methods for synthesis of AuNPs. Created by BioRender.com.

The synthesis of AuNPs can be generally categorized into different approaches: physical and chemical methods, and green synthesis (Figure 2), though certain hybrid techniques are also in use. Each method offers control over the size, shape, surface characteristics, and purity of the nanoparticles, factors that are critical for their interactions with biological systems and their effectiveness as radiosensitizers in PT.

Physical methods of AuNP synthesis rely on manipulating physical parameters such as temperature, pressure, or energy inputs. These methods are valued for producing nanoparticles with high purity, as they avoid the use of chemical reducing agents or stabilizers that may introduce impurities or toxic by-products [36]. Laser ablation, a widely utilized physical technique, involves irradiating a gold target immersed in a liquid medium with a high-energy pulsed laser, causing rapid heating and vaporization of the gold surface, followed by nucleation and growth of AuNPs in the surrounding liquid. This method provides excellent control over the size and shape of the nanoparticles by adjusting parameters like laser wavelength and pulse duration. Shorter pulses tend to yield smaller nanoparticles, while longer pulses can produce larger ones [37]. Additionally, laser ablation offers versatility in producing various shapes, including spherical, rod-like, and more complex structures such as nanostars and nanocubes [38]. The absence of chemical agents in this process results in nanoparticles of high purity, which is particularly advantageous for biomedical applications [39]. However, the cost and complexity of the required equipment make this technique less suitable for large-scale production.

Another physical method, physical vapor deposition (PVD), involves vaporizing gold atoms in a vacuum chamber via thermal evaporation or sputtering, followed by condensation on a substrate or within a gas stream to form nanoparticles [40]. PVD allows for precise control over nanoparticle size and shape by adjusting parameters like temperature and pressure [41]. Similarly to laser ablation, PVD yields highly pure nanoparticles, though it is more commonly applied to deposit nanoparticles on solid surfaces, limiting its use for creating colloidal AuNPs used in biological systems.

Mechanical techniques like ball milling can also produce AuNPs. In this method, bulk gold material is subjected to mechanical forces in a high-energy ball mill, leading to fragmentation and the formation of nanoparticles [42]. While ball milling is cost-effective and capable of producing large quantities of nanoparticles, the resulting particle size distribution is often broad, and the mechanical process may introduce impurities, which can compromise their use in biomedical applications [43].

Chemical methods of AuNP synthesis, by contrast, involve the reduction of gold salts to nanoparticles, making them a popular choice due to their scalability and fine control over particle size and shape. In the widely known Turkevich method, trisodium citrate acts as both a reducing and capping agent, converting gold ions (Au^3^⁺) into neutral gold atoms (Au^0^), resulting in spherical AuNPs with sizes ranging from 10 to 50 nm [44]. The size of the nanoparticles can be controlled by adjusting the ratio of citrate to gold salt [45]. Another approach, using sodium borohydride as a reducing agent, produces smaller nanoparticles, typically within the 2–10 nm range. In this approach, sodium borohydride reduces gold ions in solution to metallic gold, initiating nucleation and rapid growth of nanoparticles. The strong reducing power of NaBH₄ facilitates rapid reduction, leading to smaller, more uniformly sized nanoparticles due to controlled nucleation [44].

Chemical reduction methods are highly scalable and versatile, allowing for the synthesis of various AuNPs’ shapes, including spheres, rods, and cubes [46]. However, these methods may introduce impurities due to the use of reducing and stabilizing agents, necessitating further purification steps for biomedical applications.

An alternative chemical technique is seed-mediated growth, which involves first synthesizing small gold seeds through chemical reduction, and then promoting their growth in the presence of additional gold ions and a mild reducing agent [47]. This method allows for precise control over the size and shape of the final nanoparticles and is particularly effective in producing nanorods, nanocubes, and nanostars [48]. By varying the concentration of the seed solution and reducing agents, nanoparticles with sizes ranging from 5 nm to over 100 nm can be synthesized.

Green synthesis offers an environmentally friendly alternative, using natural products such as plant extracts, bacteria, or fungi to reduce gold ions [49]. Organic compounds present in these biological agents, such as polyphenols and proteins, act as reducing and stabilizing agents, resulting in nanoparticles with high biocompatibility and lower cytotoxicity [50]. This method avoids the use of toxic chemicals, aligning with sustainable and biocompatible approaches, although it may pose challenges in controlling particle size and batch reproducibility.

The scalability of green synthesis remains a pivotal concern in translating laboratory successes to industrial-scale production. While traditional chemical methods offer reproducibility and control, they often rely on hazardous chemicals that may limit their applications in medical fields. Green synthesis, on the other hand, leverages the rich biochemical diversity of natural reducing agents to produce nanoparticles. For instance, Shankar et al. demonstrated that gold nanoparticles synthesized using Azadirachta indica (neem) leaf extract not only exhibited excellent biocompatibility but also showed potential for scalable production by optimizing extraction and reaction conditions [51]. However, challenges such as batch-to-batch variability and the complexity of controlling nanoparticle size and morphology necessitate further standardization. A recent review by Karnwal et al. emphasizes the need for scalable and standardized methods to improve green synthesis reproducibility [52].

The biocompatibility of green-synthesized nanoparticles is inherently superior to those produced through chemical routes. Biocompatibility is achieved due to the capping and stabilizing effects of natural biomolecules, which render the nanoparticles less cytotoxic. For example, Rojas-Cessa et al. demonstrated the synthesis of gold nanoparticles (AuNPs) using plant extracts, emphasizing the role of natural capping agents in enhancing biocompatibility. Their study highlighted that the biomolecules from the plant acted as reducing and stabilizing agents, resulting in AuNPs with minimal cytotoxic effects and potential applications in cancer therapy [53]. The potential for clinical-grade production of green-synthesized nanoparticles is underpinned by advancements in process optimization and regulatory compliance. Ensuring Good Manufacturing Practice (GMP) compliance requires stringent control over the synthesis process, including the source of biological material, extraction methods, and reaction conditions [54]. In clinical applications, green-synthesized nanoparticles show promise due to their enhanced biological interactions. The functionalization of these nanoparticles with targeting ligands or therapeutic agents further expands their utility. For instance, Wu et al. reported the synthesis of gold nanoparticles decorated with epigallocatechin-3-gallate (EGCG) nanospheres. This green synthesis approach provided enhanced biocompatibility and stability, while functionalization with EGCG improved the nanoparticles’ targeting ability and photothermal conversion efficiency, making them suitable for applications in cancer therapy [55]. Moreover, studies have demonstrated the feasibility of integrating green synthesis into large-scale production pipelines. For example, Gurunathan et al. developed a green chemistry approach for synthesizing biocompatible gold nanoparticles (AuNPs) using natural biomaterials. Their study highlighted the scalability of the method, which optimized reaction parameters such as pH, temperature, and biomaterial concentration to produce AuNPs with consistent size and morphology. This approach demonstrated significant potential for industrial-scale production while maintaining biocompatibility, making it suitable for biomedical applications [56]. In conclusion, the green synthesis of nanoparticles offers a sustainable and biocompatible alternative to conventional methods, with significant potential for scalability and clinical-grade production. Continued research into optimizing synthesis conditions, coupled with advancements in process standardization and functionalization techniques, will be instrumental in realizing the full potential of green-synthesized nanoparticles in biomedicine.

Finally, electrochemical synthesis involves reducing gold ions onto the surface of an electrode by applying a controlled voltage [57]. This method offers precise control over nanoparticle size and shape by adjusting parameters like current density and the composition of the electrolyte solution [58]. Electrochemical synthesis is advantageous for producing highly pure nanoparticles, although the requirement for specialized equipment and the challenge of scaling up production may limit its broader application.

Each method presents unique benefits and limitations, and the choice of synthesis technique depends on the specific application and desired properties of the AuNPs, particularly in biomedical contexts where purity, biocompatibility, and control over size and shape are critical factors.

The unique physical and chemical properties of AuNPs make them ideal candidates for enhancing PT. Their small size, large surface area, and biocompatibility, coupled with the ability to modify surface chemistry, allow AuNPs to selectively amplify radiation effects in tumor tissues while minimizing damage to healthy cells [24,59,60].

Size and shape are crucial parameters that influence the interaction of AuNPs with both biological systems and proton beams. AuNPs typically range from 1 nm to over 100 nm in size. Smaller nanoparticles, particularly those under 10 nm, can penetrate deeper into tissues and cells, even crossing biological barriers like the blood–brain barrier [61]. However, larger nanoparticles, while often having greater cellular uptake, face a higher risk of immune clearance.

The optical properties of AuNPs, particularly their localized surface plasmon resonance (LSPR), are another critical factor. LSPR refers to the collective oscillation of electrons at the surface of the nanoparticles when exposed to light, leading to strong absorption and scattering at specific wavelengths [62]. This property is highly sensitive to changes in particle size and shape, with smaller nanoparticles absorbing light within the visible spectrum, while nanorods exhibit resonances in the near-infrared region [63]. These optical properties make AuNPs suitable for applications in deep tissue imaging and photothermal therapy, in addition to PT, where LSPR can enhance the efficiency of proton interactions by increasing the yields of secondary electrons and ROS, leading to localized damage in cancer cells [64,65,66].

Thermal conductivity is another significant physical property of AuNPs. Gold’s excellent thermal conductivity can be exploited in hyperthermia treatments, where externally applied heat, often through lasers, is converted by the nanoparticles into thermal energy [67]. Once concentrated in tumor tissues, this heat can ablate cancer cells directly or sensitize them to proton radiation, improving treatment outcomes.

The chemical properties of AuNPs are equally important, particularly their versatile surface chemistry. The surface of AuNPs can be easily modified with various ligands, drugs, and targeting molecules, enhancing stability, biocompatibility, and targeting efficiency—key factors in PT [68]. Gold’s affinity for sulfur- and nitrogen-containing groups allows for easy attachment of different molecules, enabling functional flexibility. This flexibility allows for conjugation with drugs, targeting ligands, and stabilizing agents, facilitating precise delivery to tumor sites [69].

In biological applications, surfaces are often modified to resist aggregation and improve colloidal stability. For instance, attaching polyethylene glycol (PEG) to the surface creates a steric barrier that prevents immune system clearance and prolongs circulation time in the body, enhancing tumor accumulation via the enhanced permeability and retention (EPR) effect [70,71].

## 4. Mechanisms of Action of AuNPs

### 4.1. Cellular Interaction of AuNPs

AuNPs are known for their ease of preparation [72] and ability to control their shape and size, offering excellent biocompatibility and optical properties such as surface-enhanced Raman scattering (SERS), two-photon luminescence (TPL), and surface plasmon resonance (SPR), making them highly promising for biomedical applications like drug and gene delivery, hyperthermia, imaging techniques, and biocatalysis. The shape, size, and surface chemistry of AuNPs are critical in influencing their physiological behavior, affecting targeting, blood circulation, distribution, metabolism, translocation, elimination, and inflammation in vivo, as well as cellular pathways in vitro, which underscores the need to evaluate both short- and long-term effects of AuNPs to predict nanotoxicity and ensure their effectiveness and safety in biomedical contexts [73,74,75].

**Figure 3:**
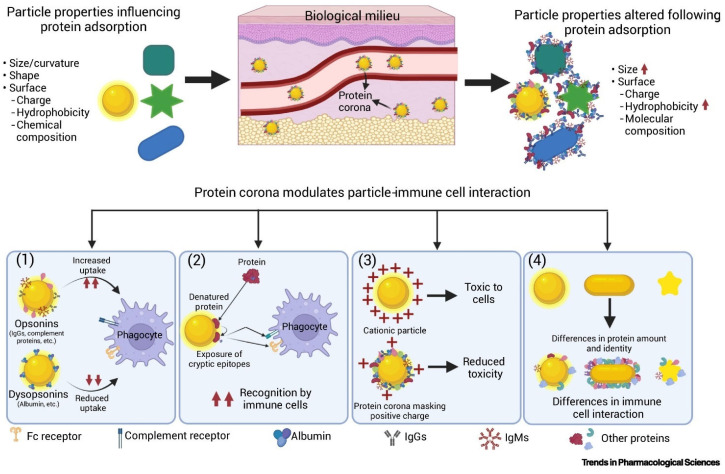
Influence of the protein corona on particle–immune cell interactions. Reproduced from Sarma et al., 2022 [77], with permission from Elsevier.

AuNPs enter biological fluids and proteins and biomolecules rapidly adsorb onto their surface, forming a “protein corona” that reduces surface free energy and can alter the structure and function of the proteins, potentially lowering targeting efficacy and inducing variable cellular responses like inflammation, increased lysosomal permeability, apoptosis, and caspase-related pathway activation [76] (Figure 3).

This protein corona alters the distribution of AuNPs in tissues and organs, highlighting the importance of analyzing AuNP–protein interactions for understanding biological outcomes. Inside the human body, AuNPs can enter through routes such as intravenous injection, inhalation, oral administration, and dermal exposure, and they interact with molecules like lipids, polysaccharides, nucleic acids, and proteins, forming a solid–liquid interface that modulates their biological effects [78].

Upon entering cells, AuNPs interact with various cell types like macrophages, lymphocytes, endothelial cells, and monocytes, influencing oxidative stress, adhesion, proliferation, apoptosis, differentiation, and inflammatory responses. Their nanometer scale enables interactions with cellular structures and organelles, rendering them particularly suitable for advanced analytical techniques to reveal dynamic biological effects. AuNP internalization occurs via processes such as phagocytosis and receptor-mediated endocytosis, whereby nanoparticle–protein complexes are recognized by membrane receptors and internalized. Depending on their size, AuNPs can localize to the cytoplasm or nucleus; for example, smaller AuNPs (2–6 nm) can penetrate the nucleus, while those larger than 15 nm remain confined to the cytoplasm [79].

Key factors influencing the biological behavior of AuNPs include their size, shape, surface modifications, aspect ratio, and charge, which significantly impact their interactions with cell membranes and internalization mechanisms. These processes are crucial for the safety and therapeutic efficacy of AuNPs in biomedicine, where ongoing studies focus on clarifying cellular mechanisms and optimizing nanomaterial properties to enhance their biomedical utility [80].

Additionally, methodological advancements in high-throughput screening (HTS), omics approaches (genomics, proteomics, metabolomics), and bioinformatics are critical for predicting cellular risks and unraveling the complex molecular signaling networks involved in AuNP interactions.

Finally, developing in situ and real-time analytical techniques capable of capturing the interfacial interactions between AuNPs, proteins, and membrane structures is essential for advancing the understanding of nanoparticle behavior, ensuring that future AuNP applications are both functional and biocompatible in clinical settings.

AuNPs have been extensively studied for their biocompatibility, ease of synthesis, and potential for surface functionalization to target tumors [81,82,83]. These AuNPs, when conjugated with chemotherapeutic agents such as doxorubicin, have demonstrated enhanced cytotoxic effects on cancer cells, while minimizing damage to healthy tissues, reducing overall treatment toxicity [84].

As researchers explore the therapeutic possibilities of AuNPs, attention has shifted to their application in radiotherapy, particularly for enhancing dose localization and minimizing adverse effects.

PT, a highly targeted form of radiotherapy, has also been identified as a promising area for the integration of AuNPs [85,86]. Due to their physicochemical properties, AuNPs can improve the therapeutic efficacy of PT by interacting with biological systems at the molecular level.

The following sections will provide an overview of the mechanisms by which AuNPs can enhance radiosensitivity in PT and their potential clinical applications.

Upon introduction into the body, AuNPs interact with cellular structures such as the plasma membrane, cytoplasm, and nucleus [87,88,89]. These interactions are critical for understanding how AuNPs enhance therapeutic outcomes. The biodistribution and internalization of AuNPs depend heavily on their size, shape, and surface charge, which influence their ability to reach target tissues and cells. Nanoparticles ranging between 10 and 50 nm are internalized more efficiently through endocytosis than those outside this range [90]. Surface charge is also a crucial determinant, as positively charged AuNPs are more readily internalized by cells due to electrostatic interactions with negatively charged cellular membranes [91]. Furthermore, surface functionalization with specific ligands, such as peptides, antibodies, or small molecules, can direct AuNPs to receptors that are overexpressed on tumor cells, improving their specificity and uptake [92,93,94]. Once inside the cell, AuNPs must escape endosomal compartments to reach their intended intracellular targets. Post-endocytosis, AuNPs are typically trafficked to lysosomes, where they may be degraded or sequestered [95]. For AuNPs to exert their radiosensitizing effects, they must successfully navigate the intracellular environment, escaping degradation and reaching the cytoplasm or nucleus [15]. This is particularly important for nuclear-targeted therapies, which aim to increase DNA damage through radiation. For instance, oligonucleotide-functionalized AuNPs have been shown to target telomerase-positive cancer cells, increasing DNA double-strand breaks (DSBs) and enhancing radiosensitivity [96].

### 4.2. Mechanisms of Enhanced Radiosensitivity

AuNPs enhance the radiosensitivity of tumor cells during PT through complex, multifactorial mechanisms involving both physical and biological processes at the cellular and molecular levels. When exposed to proton radiation, AuNPs significantly increase the local energy deposited within the cell, leading to an enhancement of the dose absorbed by tumor tissue [97]. This increase in local dose, often referred to as *dose enhancement*, increases the biological effectiveness of radiation.

Several studies have confirmed the dose-enhancement effects of AuNPs in PT. For instance, Zhang et al. demonstrated how gold nanomaterials enhance radiosensitivity by increasing local radiation effects in tumor cells and interfering with cellular repair mechanisms [70]. Similarly, Wang et al. highlighted the efficacy of AuNPs in glioma cells, where they were shown to inhibit the TRAF6/NF-κB pathway, thereby reducing the expression of CCL2, a key factor in tumor cell survival and resistance to radiotherapy. These findings suggest that AuNPs not only increase the physical dose delivered to the tumor but also disrupt the biological processes that enable tumor cells to recover from radiation-induced damage [98].

At the molecular level, AuNPs enhance radiosensitivity through three primary mechanisms: (1) augmentation of DNA damage, (2) increased production of reactive oxygen species (ROS), and (3) disruption of cellular repair mechanisms. First, AuNPs play a critical role in amplifying DNA damage by enhancing energy deposition near the DNA, leading to more extensive localized damage, which is harder for cells to repair. Additionally, AuNPs stimulate the production of ROS, which contribute to cellular damage by inducing oxidative stress and disrupting essential biomolecules, such as lipids, proteins, and nucleic acids. Finally, AuNPs interfere with cellular repair mechanisms, further hindering the ability of cancer cells to recover from radiation-induced damage. Together, these effects synergistically increase the biological impact of radiation therapy, enhancing tumor control. In the following sections, we will explore these mechanisms in more detail.

#### 4.2.1. DNA Damage Augmentation

AuNPs enhance DNA damage during PT through both direct and indirect mechanisms, making them highly effective radiosensitizers at the molecular and cellular levels. The direct mechanism is primarily due to the physical properties of gold, with its high atomic number (Z = 79) allowing it to efficiently absorb energy when exposed to proton irradiation [99]. This interaction leads to the emission of secondary particles, such as Auger electrons, which have an extremely short range, typically on the order of nanometers [86].

**Figure 4:**
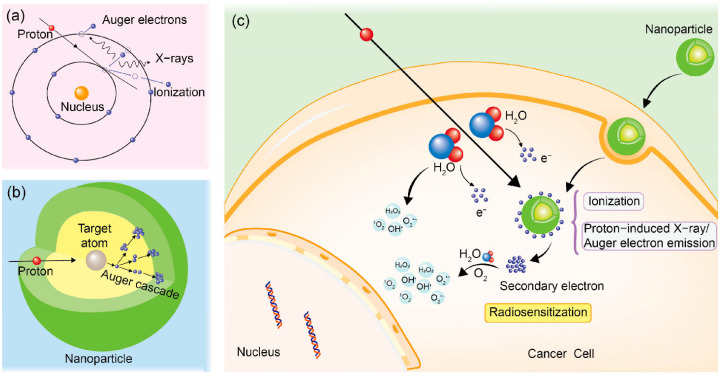
Mechanisms of nanoparticle radiosensitization in PT. (**a**) Processes of ionization and emission of proton-induced X-rays and Auger electrons resulting from interactions between protons and target atoms. (**b**) Process of Auger cascade. (**c**) Illustration of increased physical dose deposition and enhanced radiolysis in cancer cell with presence of nanoparticles. Reproduced with permission from Ma et al., *Cells*, published by MDPI, 2024 [7].

In cancer therapy, the Auger effect is utilized for its ability to cause highly localized damage to cancer cells. When high atomic number elements (e.g., AuNPs) are introduced into tumors and exposed to ionizing radiation, the Auger effect generates secondary electrons that create dense ionization at a small volume. The effect is particularly effective when nanoparticles are targeted to accumulate near DNA. This precision makes the Auger effect a promising tool for enhancing radiotherapy efficacy (Figure 4). These low-energy electrons release energy in a highly localized manner around the AuNPs, particularly near DNA molecules [100]. This localized energy deposition results in the creation of multiple types of DNA lesions, including single-strand breaks (SSBs) and double-strand breaks (DSBs).

SSBs occur when the phosphate backbone of the DNA is damaged on only one strand of the double helix [101]. Although these breaks are more easily repaired by the cell through the base excision repair (BER) pathway, AuNPs increase the frequency of these lesions [102].

Furthermore, when multiple SSBs occur in close proximity due to the dense ionization from secondary electrons, they can escalate into more severe lesions, such as DSBs. DSBs, which involve breaks on both DNA strands, are particularly lethal because they disrupt the continuity of the genetic material, leading to genomic instability, mutations, or cell death if not properly repaired [103,104]. AuNPs increase the likelihood of DSBs forming due to their ability to concentrate ionization events in specific regions, creating complex DNA damage that is difficult for the cell to repair.

For instance, Zhao et al. demonstrated that AuNPs possess a significant radiosensitizing effect. Using models based on Monte Carlo simulations with the Geant4-DNA toolkit, the study quantified DNA damage, such as double-strand breaks (DSBs), caused by irradiation with photons at different energies (100 keV and 250 kVp). Simulations revealed that AuNPs, especially large ones (up to 100 nm), significantly increase the number of DSBs, up to 64% in some configurations, due to their ability to concentrate ionization events in the vicinity of the cell nucleus [105].

The accumulation of such damage leads to significant genomic instability and an elevated risk of cell death, positioning AuNPs as highly effective radiosensitizers. However, the indirect effects of AuNPs, particularly through the production of reactive oxygen species (ROS), further amplify their radiosensitizing capabilities. The generation of ROS plays a critical role in amplifying oxidative stress within the cell, leading to additional molecular damage that complements the direct ionization effects. Therefore, the dual contribution of both direct and indirect mechanisms positions AuNPs as promising agents in enhancing the efficacy of PT.

#### 4.2.2. Production of ROS and Cellular Repair Mechanisms Disruption

The generation of reactive oxygen species (ROS) is a crucial mechanism through which AuNPs enhance the radiosensitivity of tumor cells during PT [106]. ROS are chemically reactive molecules containing oxygen, such as hydroxyl radicals (·OH), superoxide anions (O_2_^−^), and hydrogen peroxide (H_2_O_2_) [107]. These molecules play a central role in mediating oxidative stress and causing damage to cellular components, particularly DNA, proteins, and lipids. The ability of AuNPs to amplify ROS production during PT significantly contributes to the overall cytotoxic effects on cancer cells [108].

When AuNPs are introduced into tumor cells and subjected to proton radiation, they enhance the production of ROS through a series of direct and indirect interactions with the cellular environment [109].

PT induces radiolysis of water molecules in the cellular cytoplasm, leading to the formation of highly reactive hydroxyl radicals (·OH), which initiate oxidative damage [110]. The addition of AuNPs enhances this process due to their high atomic number, which increases energy absorption from radiation.

This absorbed energy results in the emission of secondary electrons, such as Auger electrons, which further interact with nearby water molecules, amplifying the production of ROS [111]. For instance, Tsai et al. demonstrated that AuNPs enhance the generation of ROS when irradiated by Cs-137, significantly improving radiotherapy outcomes. Their findings showed that AuNP uptake increased oxidative damage to cellular organelles, such as mitochondria and the cytoskeleton, leading to enhanced tumoricidal effects. With a radiosensitization enhancement factor of 1.29, AuNPs present a promising strategy to improve the efficacy of Cs-137-based radiotherapy, particularly for radioresistant tumors [112].

The surface chemistry of AuNPs is pivotal in enhancing their catalytic activity, especially under proton radiation, where their interaction with radiation leads to increased ROS production and intensified cytotoxic effects. Studies have demonstrated that the surface properties of AuNPs, such as the presence of ligands and the specific surface area, play a crucial role in determining the extent of ROS yield. For example, Johny et al. demonstrated how ligand-free AuNPs with larger surface areas significantly amplify ROS production in PT, showing a clear link between surface chemistry and radiosensitization [113].

ROS also induce extensive damage to lipids, proteins, and DNA within cells. Oxidative damage to lipids compromises membrane integrity by inducing lipid peroxidation, which leads to significant structural changes such as bilayer thinning, increased membrane tension, and altered fluidity. These changes result in heightened membrane permeability and ultimately promote cell death. Lipid peroxidation typically involves the formation of reactive oxygen species (ROS) that oxidize polyunsaturated fatty acids within membrane phospholipids. This oxidative stress can trigger cell death mechanisms like ferroptosis, where the accumulation of peroxidized lipids at the plasma membrane activates ion channels such as Piezo1, increasing cation permeability and disturbing ionic homeostasis. The resulting disruption of membrane function, particularly the increased permeability to ions like Na^+^ and Ca^2+^, leads to cellular swelling, loss of membrane potential, and eventual cell lysis [114,115].

Proteins exposed to reactive oxygen species (ROS) frequently undergo denaturation or misfolding, which results in functional impairments that contribute to cellular dysfunction. This process is particularly damaging as ROS can oxidize amino acid residues, causing irreversible changes to protein structure, such as the formation of disulfide bonds, carbonylation, and crosslinking. These oxidative modifications compromise protein folding, often leading to aggregation or the inability of the cell’s degradation machinery, such as the proteasome or autophagy pathways, to eliminate damaged proteins efficiently. Misfolded proteins that accumulate in the cell disrupt normal cellular functions, which is linked to a variety of age-related diseases, including neurodegenerative disorders and cardiovascular diseases [116,117].

However, the most critical consequence of ROS in PT is the damage inflicted on DNA. ROS oxidize DNA bases, particularly guanine, generating 8-oxo-7,8-dihydroguanine (8-oxoG), a mutagenic lesion [118]. ROS also induce single-strand breaks (SSBs) and double-strand breaks (DSBs) in DNA, with DSBs being particularly cytotoxic due to the difficulty in accurate repair, often resulting in genomic instability, mutations, or cell death [119].

In PT, the addition of AuNPs significantly amplifies the production of ROS, leading to more extensive oxidative damage compared to PT alone. AuNPs increase the number of secondary electrons, which interact with water molecules to generate ROS, as demonstrated by Lo et al. [111]. In their study, AuNP-loaded tumor cells irradiated by a proton beam in the spread-out Bragg peak region showed a marked increase in ROS production, resulting in enhanced cytotoxicity due to mitochondrial dysfunction and cellular damage. Similarly, Tabatabaie et al. observed that AuNPs intensified mitochondrial stress by increasing ROS levels in cancer cells, which peaked at around 4 Gy radiation doses. This oxidative stress accelerates cell death, enhancing the therapeutic efficiency of PT [120].

The synergy between ROS production and PT, combined with the direct ionizing effects of radiation, significantly enhances cancer treatment efficacy. The ability of AuNPs to modulate ROS production presents opportunities for optimizing nanoparticle design, enabling more targeted and effective cancer therapies.

## 5. Energy Deposition and Dose Enhancement

AuNPs have been identified as a mechanism for enhancing the effectiveness of radiation therapy doses, and one of the key mechanisms is the generation of secondary electrons when ionizing radiation interacts with AuNPs. These high-energy electrons, capable of traveling significant distances, release energy in the areas surrounding cancer cells, leading to a higher concentration of energy and an increased radiation dose near the AuNPs [121,122]. A significant mechanism proposed by Xie et al. is the Auger cascade phenomenon, where high-energy photons or electrons hitting AuNPs can excite or ionize gold atoms, resulting in the emission of Auger electrons with a high linear energy transfer (LET) [123,124]. These Auger electrons are particularly efficient at depositing energy locally, causing all the effects described in the previous paragraph.

Understanding these physical mechanisms is crucial for developing strategies to maximize the therapeutic benefits of AuNPs while minimizing potential side effects, and by conducting a detailed analysis of how these particles affect energy deposition and ROS production, researchers can refine treatment techniques and improve the precision of radiotherapy. Experimental studies, along with Monte Carlo simulations, play a critical role in grasping the complex interactions between AuNPs and radiation, thus helping to develop innovative strategies in radiotherapy, which holds great promise for improving cancer treatment outcomes [125]. DNA damage caused by these processes can be categorized into indirect damage, which results from chemical reactions between DNA molecules and free radicals, and direct damage, stemming from physical interactions. This damage includes both single-strand breaks (SSBs) and double-strand breaks (DSBs), with Monte Carlo simulations being instrumental in calculating the dose enhancement effect at microscopic and nanoscopic levels by modeling the interaction of radiation with objects like AuNPs [126]. These simulations, aiming to quantify the increase in radiation dose near the nanoparticles, follow a process involving several stages, including geometric modeling, simulating particle interactions, calculating energy deposition, estimating dose enhancement, and finally measuring and analyzing the deposited energy, which is then validated and subjected to sensitivity analysis to ensure both accuracy and reliability.

The DNA damage caused by increased energy deposition in the presence of AuNPs can be quantified through the dose enhancement ratio (DER), with the size of AuNPs playing a significant role in this ratio. In certain clinical scenarios and specific conditions, larger particles may offer advantages, yet further studies are needed to explore the complexities of the interactions between AuNP size, photon beams, and dose enhancement effects in radiotherapy. It has been observed that the DER increases with nanoparticle size, as larger AuNPs contain more gold atoms, which leads to an increase in photon interactions and greater secondary electron generation, and while some studies suggest this increase is due to greater energy accumulation in the DNA, it is also important to consider that, as nanoparticle size increases, self-absorption becomes more relevant [127]. Studies confirm that larger AuNPs tend to exhibit higher levels of electron self-absorption, meaning that despite the initial increase in the DER with larger AuNPs, this self-absorption can reduce the DER, making it crucial to factor in this effect when determining the optimal size of AuNPs for radiotherapy applications. Furthermore, the reduction in the DER with increasing photon beam energy aligns with findings that show how higher photon energies decrease the efficacy of photoelectric interactions due to lower cross-sections [128].

Bardane et al. used Monte Carlo simulations to examine the dose enhancement effect caused by AuNPs with GATE-9 and the Geant4 toolkit, which provided in-depth insights into the impact of dose enhancement at nano- and microscopic levels, clarifying the mechanisms involved and the amplified response to radiation in the presence of AuNPs [129]. This study confirmed that AuNPs have the potential to improve the therapeutic effects of cancer radiotherapy by selectively increasing the radiation dose delivered to tumor tissues due to their strong interaction with photons and their ability to produce secondary electrons.

## 6. Clinical Applications and Trials

### 6.1. Preclinical Studies

Preclinical studies investigating the potential of AuNPs to enhance PT have provided promising insights into their efficacy as radiosensitizers (Table 2). These studies, conducted both in vitro and in vivo, have focused on understanding the biological mechanisms underlying AuNPs-mediated radiosensitization and optimizing nanoparticle characteristics such as size, shape, concentration, and surface functionalization [59].

To develop effective radiosensitizers based on AuNPs, it is crucial to optimize their parameters and consider the use of multifunctional platforms. These platforms can combine radiosensitization with imaging, thermal capabilities, and drug delivery, offering theranostic applications. Furthermore, to enhance the in vivo performance of AuNPs, it is important to consider additional factors such as radiation energy, fractionation, and biological responses to radiation [85].

**Figure 5:**
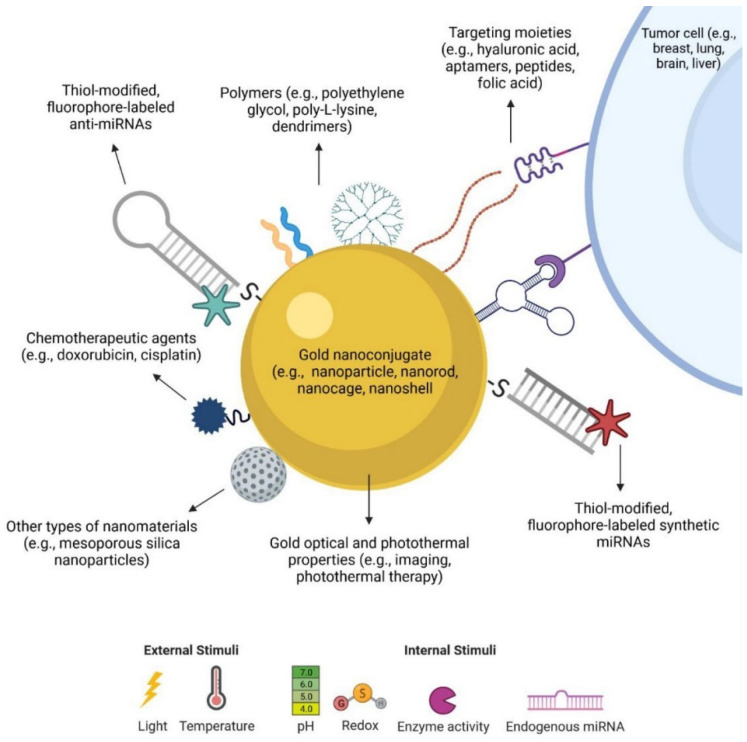
A diagram illustrates various ways AuNPs can be modified for use in developing miRNA-based therapies. AuNPs come in various shapes (such as spheres, rods, cages, and shells) and possess unique properties that can be customized by attaching different functional groups. These include oligonucleotides, like anti-miRNAs and synthetic miRNAs, which may themselves be modified with thiol groups or fluorescent tags. Additionally, the nanoparticles can be conjugated with polymers to enhance stability and biocompatibility, targeting ligands to increase specificity for cancer cells, chemotherapeutic drugs, or even other nanomaterials. Furthermore, many of these functional molecules can be designed to be released in response to specific external or internal triggers, such as light exposure or changes in pH. Reproduced with permission from Sousa and Conde, ACS, 2022, licensed under cc-by-nc-nd 4.0 [139].

The use of AuNPs in tumor tissue improves image contrast during the diagnostic phase, facilitating tissue preparation for radiotherapy. Furthermore, these nanoparticles can be chemically modified with complex molecules such as polyethylene glycol, amine groups, carboxyl groups, peptides, DNA, RNA, and antibodies [134] (Figure 5).

Kim et al. demonstrated that proton treatment combined with AuNPs in mice with subcutaneous CT26 colorectal adenocarcinoma led to a more than 50% increase in one-year survival compared to proton irradiation alone [135]. Similarly, in vitro studies have shown that AuNPs can enhance the sensitivity of cancer cells to PT. When AuNPs are internalized by cancer cells and then exposed to protons, there is a significant increase in DNA damage compared to PT alone [86].

PT itself offers high precision in targeting malignant tumors due to the Bragg peak effect, allowing it to deliver higher radiation doses while sparing surrounding healthy tissue. Recent advancements in PT have included sophisticated imaging systems, improved beam control, and advanced scanning methods like pencil beam scanning, all of which optimize the precision of treatment [136]. Polf et al. examined the effects of various treatments on DU145 human prostate cancer cells, comparing those treated with both AuNPs and internalizing phages, phages alone, and no treatment. Following irradiation, clonogenic assays indicated that the targeted internalization of AuNPs significantly enhances the efficacy of proton radiotherapy. The cell survival curves revealed that the proton–nanoparticle interaction increases ionization density within the cells, leading to higher rates of cell death. Cells treated with AuNPs displayed a notable reduction in survival [137].

AuNPs serve both as radiosensitizers—weakening cancer cells before irradiation—and as radio-enhancers by amplifying the physical dose of radiation [54]. Tudda et al., using time-lapse light microscopy and electron microscopy, found no adverse effects on cell viability when AuNPs were used alone. However, with irradiation at doses up to 2 Gy, cell survival curves indicated increased cell death in the presence of AuNPs compared to irradiation without them [138].

A key parameter is the choice of synthesis. As explained by Torrisi et al., the synthesis of AuNPs using the laser method is preferred. This approach yields a purer solution without the need for additional chemical agents, unlike traditional chemical synthesis. This method produces nanoparticles with a uniform size between 5 and 10 nm, making it easier for them to cross the membrane and enter the intracellular fluid. In addition, the spherical shape of the nanoparticles helps reduce friction forces during their diffusion movement [134].

However, despite numerous studies, the lack of key data and the variability of experimental conditions make it difficult to obtain definitive conclusions. It is crucial to accurately characterize AuNPs (including parameters such as size, coating, shape, and gold content in cells or tumors) to better understand their role in physical and chemical enhancement. It is recommended to establish a set of cell lines to compare different DNA repair capabilities and radiosensitivity. It is important to study the effect of time between AuNP incubation and radiation exposure, an aspect that is often overlooked, and of developing new techniques to correlate biological dysfunction with AuNP uptake in cell subpopulations. These combined parameters may optimize the therapeutic efficacy of AuNPs by enhancing physical, chemical, and biological effects, and guide the design of future in vivo experiments [54].

In this regard, it was shown in the work of Liu et al. that, both in vitro and in vivo, AuNPs significantly increase the production of free radicals and reactive oxygen species (ROS) in tumor cells subjected to proton radiation [140]. In vivo experiments in mouse models showed a significant reduction in tumor growth in groups treated with AuNPs and protons, compared to those exposed to protons alone [141].

A study by Lo et al. explored the radiosensitizing effects of AuNPs by examining their role in enhancing reactive oxygen species (ROS) production in tumor cells exposed to proton beam radiation, specifically at the spread-out Bragg peak. Through in vitro studies, they demonstrated that AuNPs significantly increase ROS levels, which contributes to greater DNA damage, particularly double-strand breaks, thereby amplifying the efficacy of PT in targeted cancer treatments [111].

Similarly, in vivo research by Chithrani et al. highlighted that tumor-bearing mice treated with PT and AuNPs exhibited significantly delayed tumor growth and increased apoptosis compared to controls receiving only PT [95]. Furthermore, studies have demonstrated that the shape of AuNPs plays a crucial role in their radiosensitizing effect, with spherical nanoparticles often showing greater efficacy compared to other shapes like rods or stars, likely due to more efficient cellular uptake and interaction with proton-induced ionization events [142].

Moreover, surface modifications such as PEGylation were shown to improve the biodistribution and tumor uptake of AuNPs. For instance, Liu et al. demonstrated that PEGylated AuNPs significantly enhanced the radiosensitivity of the cancer cells in vitro. Their study showed that when EMT-6 and CT26 cancer cells were treated with PEG-AuNPs and then exposed to various radiation sources, including protons, there was a marked decrease in cell survival rates. The PEG modification improved nanoparticle stability and tumor uptake, facilitating greater localized radiation dose deposition and increased DNA double-strand breaks, thereby amplifying cell death [143].

Additionally, optimizing the size of AuNPs is crucial for maximizing the efficacy of PT. Research demonstrates that smaller AuNPs, typically under 10 nm, penetrate cells more readily and distribute more uniformly within tumor tissues, improving their capacity to enhance local dose deposition during proton irradiation [144,145].

Beyond size, the concentration and dose of AuNPs are crucial parameters. Balancing the concentration and dose of AuNPs is essential for therapeutic applications, as excess levels lead to cellular toxicity without proportional benefits [146]. Studies report that low doses can minimize adverse effects, maintaining cellular integrity [73]. For example, AuNPs used in radiosensitization increased efficacy with minimal side effects when carefully dosed [147]. Optimizing dosing reduces toxic reactive oxygen species while leveraging therapeutic effects [148].

Another key aspect in enhancing the therapeutic efficacy of AuNPs is their biodistribution and pharmacokinetics. Effective targeting strategies are essential to maximize tumor accumulation while minimizing off-target effects, ensuring a focused and efficient delivery to cancerous cells [149]. Despite their advantages, challenges with AuNP clearance and long-term biocompatibility persist, as they tend to accumulate in organs like the liver and spleen, raising concerns about chronic toxicity [150]. Furthermore, the potential for immunogenic responses, such as immune modulation depending on AuNP surface chemistry or morphology, highlights the need for further studies on their interactions with the immune system [49].

Despite these promising preclinical results, more extensive studies are necessary to better elucidate the long-term toxicity, clearance pathways, and potential immunogenic responses associated with AuNPs’ use in PT. As the field progresses toward clinical applications, it will be essential to integrate findings from diverse biological models and develop standardized protocols for AuNPs’ administration, ensuring safe and effective use in human patients.

### 6.2. Mitigation Strategies for Long-Term Toxicity of AuNPs

The long-term toxicity of AuNPs is a critical factor in their biomedical application, particularly in radiotherapy. Despite their recognized biocompatibility, studies indicate that AuNPs tend to accumulate in vital organs such as the liver, spleen, and kidneys after prolonged use, leading to potential inflammatory and fibrotic responses due to the inability of the body to metabolize or excrete them efficiently [151]. This accumulation is influenced by the size, shape, and surface charge of the nanoparticles, with smaller particles (<10 nm) showing greater potential for renal clearance compared to larger counterparts [152]. However, particles optimized for tumor targeting, typically ranging between 10 and 50 nm, exhibit reduced clearance and higher organ retention, necessitating further investigation [153]. To mitigate these adverse effects, researchers have focused on developing biodegradable coatings and hybrid nanoparticles that enhance biocompatibility and clearance. Natural polymer coatings such as PEG and chitosan have demonstrated efficacy in reducing nanoparticle aggregation and improving circulation time, while also facilitating immune evasion [154]. For instance, PEGylated AuNPs have shown improved biodistribution and reduced uptake by macrophages, as highlighted by studies demonstrating enhanced tumor targeting without significant off-target toxicity [150]. Similarly, hybrid nanoparticles, which integrate AuNP cores with biocompatible shells like mesoporous silica or iron oxide, offer dual benefits of imaging contrast and controlled drug release while minimizing toxicity [155]. Another approach involves optimizing nanoparticle size and surface chemistry to ensure rapid excretion. Nanoclusters below 5 nm have shown promising results in preclinical trials due to their efficient renal clearance, though their radiosensitization efficacy may be reduced compared to larger counterparts [156]. Additionally, the use of ligands such as antibodies and peptides for active targeting can enhance tumor specificity and reduce systemic exposure [157]. Overall, the potential of AuNPs as radiosensitizers in radiotherapy remains promising, but long-term toxicity concerns highlight the importance of further refining nanoparticle design. Ongoing studies on hybrid configurations and biodegradable coatings aim to balance therapeutic efficacy with biocompatibility, promoting safer clinical applications of AuNPs [158].

### 6.3. Challenges in Clinical Translation

The challenges in the clinical translation of AuNPs for enhancing PT are diverse, encompassing dosimetry, safety, regulatory, and manufacturing considerations. This section explores the state-of-the-art in these challenges, drawing on the recent literature to highlight ongoing research efforts and unresolved issues.

Achieving precise dosimetry to quantify the dose enhancement effects (DEEs) provided by AuNPs is a critical challenge in radiation therapy, particularly at cellular and tissue levels. The DEE achieved by AuNPs is influenced by several factors, including particle size, concentration, localization within cellular structures, and the type and energy of radiation used [159].

Recent studies focus on understanding how AuNPs influence the dose enhancement in radiotherapy, with a particular interest in experimental validation and computational modeling. A recent study by Huynh and Chow investigated the interaction between AuNPs and proton beams using Monte Carlo simulations, observing that dose enhancement ratios (DERs) increase significantly with decreased proximity between AuNPs and DNA, as well as lower proton energies [160]. Similarly, Lima et al. analyzed dose enhancement factors (DEFs) using electron spin resonance, noting that the DEF is highest with lower radiation doses and specific AuNP distributions, particularly in nanocomposites with materials like monosodium glutamate [161]. These studies highlight the importance of precise dosimetric techniques and refined computational models for effective AuNP utilization in clinical radiotherapy applications.

Another significant concern in clinical translation is safety, specifically regarding the biodistribution and potential toxicity of AuNPs. These issues arise from AuNPs’ high stability and tendency to accumulate in the reticuloendothelial system (RES), which includes organs such as liver, kidney, heart, and brain, potentially leading to toxicity and long-term side effects [162]. Research indicates that nanoparticle characteristics, such as size, surface chemistry, shape, and the presence of specific coatings, significantly impact biodistribution patterns. For example, Zhou et al. have found that AuNPs under 5 nm in diameter are more likely to bypass the liver and spleen and be eliminated via renal excretion, highlighting how strategic design modifications can improve clearance rates [163]. Overall, while AuNPs hold substantial potential for enhancing cancer treatments, optimizing their biodistribution and minimizing off-target toxicity remain critical for safe clinical use.

Finally, the clinical translation of AuNP-based therapies presents considerable regulatory challenges that must be addressed to ensure their safe and effective use in clinical settings. A pivotal aspect of this process is adherence to Good Manufacturing Practice (GMP) guidelines, which govern every stage of pharmaceutical production. These guidelines encompass stringent requirements for facility design, environmental controls, equipment validation, and comprehensive documentation protocols to ensure consistency and reproducibility of nanoparticle formulations. This is particularly important for AuNP-based therapies, as their unique physicochemical properties, such as particle size, morphology, and surface functionalization, can significantly influence their biological behavior and therapeutic efficacy. For example, studies have demonstrated that slight variations in AuNP surface coatings can drastically alter biodistribution and immune recognition, underscoring the importance of strict quality control measures during production [164]. Also, Zhang et al. highlight that different AuNP formulations require precise control over particle properties to ensure predictable biodistribution and pharmacokinetics, which are crucial for achieving targeted therapeutic effects. They note that challenges such as incomplete in vivo excretion and organ accumulation require a high level of precision in synthesis to mitigate potential long-term toxicity issues [165]. Thus, achieving reproducibility and compliance with regulatory standards is fundamental to moving AuNP-enhanced therapies from research into routine clinical practice.

Regulatory agencies, such as the U.S. Food and Drug Administration (FDA) and the European Medicines Agency (EMA), mandate comprehensive data on the biodegradability and immunogenicity of AuNPs, as shown in the studies. The limited biodegradability of AuNPs presents challenges, as their prolonged presence in the body raises concerns about accumulation, which could potentially lead to long-term toxicity risks. Hua et al. demonstrated that one of the significant obstacles in translating nanomedicines to clinical settings is their biocompatibility, where the lack of rapid breakdown and clearance in the body creates a need for exhaustive toxicological assessments to identify possible long-term effects [166]. Any deviation in these parameters can affect the therapeutic efficacy and safety of the final product. Regulatory agencies require robust documentation of these processes, including stability testing under various storage conditions to verify that the nanoparticles maintain their integrity over time. A study by Tremi et al. highlighted the importance of this step, demonstrating that improperly stored AuNPs exhibited significant aggregation, which reduced their radiosensitization potential in vitro [19]. Despite progress in regulatory frameworks, challenges remain in standardizing testing methodologies across different regions. Discrepancies in regulatory requirements between the EMA and FDA can lead to inconsistencies in approval timelines and clinical trial designs. International harmonization efforts, such as those initiated by the International Council for Harmonisation of Technical Requirements for Pharmaceuticals for Human Use (ICH), aim to bridge these gaps by developing standardized guidelines for nanomedicines. Such initiatives can streamline the global approval process for AuNP-based therapies, facilitating their broader clinical adoption.

An illustrative example is the development of AuroLase^®^, a nanoshell-based AuNP designed for PTT. The manufacturer faced extensive regulatory scrutiny to demonstrate consistent production quality and to provide detailed assessments of the nanoparticle’s biodistribution, clearance rates, and potential immunogenic responses. These rigorous evaluations were crucial to address safety concerns and to meet the regulatory standards required for clinical application [167].

In summary, the path to clinical implementation of AuNP-enhanced PTT is fraught with challenges related to manufacturing precision and regulatory compliance. Ensuring GMP adherence and providing comprehensive safety data are pivotal steps in overcoming these hurdles to bring effective and safe nanoparticle-based therapies to patients.

## 7. Future Directions

### 7.1. Advances in AuNP Design and Synthesis

As described in a previous paragraph, there are different approaches for synthesizing AuNPs. In recent years, an increasing focus has been placed on green chemistry approaches, which aim to minimize the use of toxic chemicals and reduce the environmental impact by employing natural reducing and stabilizing agents derived from plants, bacteria, or other biological sources. Green chemistry methods have gained popularity, especially in biomedical and environmental applications, as they offer a more sustainable and safer route for producing nanoparticles. This approach not only addresses ecological concerns but also enhances the biocompatibility of AuNPs, which is crucial for their use in medical applications like drug delivery, imaging, and diagnostics [168].

In addition to the well-established methods, researchers are constantly optimizing the synthesis of AuNPs to achieve greater control over their shape, size, and surface characteristics. Innovations such as the use of advanced surfactants, controlled reaction environments, and fine-tuned reduction conditions have significantly improved the reproducibility and scalability of AuNP production. Furthermore, recent advancements in high-throughput screening and real-time monitoring of nanoparticle formation have enabled more precise adjustments to synthesis protocols, enhancing the quality and functionality of the nanoparticles for specific applications.

One challenge in both conventional and green synthesis methods is maintaining consistency in particle size and shape, especially when scaling up production. Researchers are addressing this by developing automated processes and employing machine learning algorithms to optimize reaction conditions in real-time. Moreover, by integrating green chemistry principles into industrial-scale processes, it is possible to significantly reduce waste and energy consumption, making the production of AuNPs more sustainable. With these optimized and reproducible methodologies, it is now easier to obtain AuNPs with diverse sizes and shapes, available from various industrial suppliers [169]. These nanoparticles come with a wide range of surface chemistries, offering flexibility for their use in a variety of fields, from medical research and treatment to environmental monitoring and catalysis. The growing understanding of AuNP synthesis continues to pave the way for more sophisticated and environmentally friendly applications, while ensuring safety and efficacy in their biomedical uses.

The sizes of AuNPs significantly influence their biological behavior and interactions with radiation. Nanoparticles smaller than 10 nm exhibit higher rates of cellular internalization and can penetrate deeper into tissues, making them suitable for treating hypoxic or dense tumor regions. However, their rapid clearance from systemic circulation may limit tumor accumulation. Larger AuNPs (20–50 nm), on the other hand, benefit from the enhanced permeability and retention (EPR) effect, which improves retention in tumors but may result in less efficient cellular uptake. Monte Carlo simulations reveal that AuNPs with a size of approximately 30 nm strike an optimal balance by maximizing dose enhancement ratios (DERs). These nanoparticles generate an increased secondary electron production near the DNA, resulting in effective tumor damage [170]. The most commonly used methods for synthesizing AuNPs include various techniques, each with specific characteristics. The Turkevich method, developed in 1951, is widely used to produce spherical AuNPs by reducing gold ions (Au^3^⁺) to metallic atoms (Au^0^) using reducing agents like citrate or UV radiation. A variant uses sodium borohydride (NaBH₄) to achieve simplified synthesis without the need for heating. The Brust method, introduced in 1994, employs a biphasic reaction in organic solvents to produce particles ranging from 1.5 to 5.2 nm, utilizing sodium borohydride and alkanethiols, with tetraoctylammonium bromide facilitating phase transfer [171]. Seed-mediated growth, primarily used for gold nanorods, involves the use of pre-synthesized particles that, through weak reducing agents, grow to the desired shape. The digestive ripening method, ideal for producing monodisperse nanoparticles, relies on heating colloidal suspensions up to 138 °C, followed by controlled cooling to modulate size distribution. The Martin method focuses on stoichiometric control to regulate the ratio of reagents and optimize synthesis, allowing AuNPs to combine with hydrophilic molecules for specific applications. The use of radiation during AuNP synthesis affects their size: higher dose rates lead to the formation of smaller nanoparticles. An increasingly relevant approach is biological synthesis, which uses living organisms like bacteria, plants, and fungi for eco-friendly AuNP production, characterized by a reduced environmental impact and high economic feasibility. Other methods employ bacteria, fungi, plants, algae, and biomolecules, each uniquely contributing to the production of nanoparticles with distinctive properties [172]. The properties of AuNPs can be further tailored through coating and functionalization. Surface functionalization further enhances the specificity and efficacy of AuNPs. Functionalization techniques, such as PEGylation, prolong systemic circulation, increase tumor uptake, and improve endosomal escape, allowing closer interaction with nuclear DNA. These surface modifications reduce immune clearance and enable selective targeting of tumor tissues. Monte Carlo models quantitatively demonstrate that the DER is maximized when nanoparticles are located within 100 nm of the cell nucleus, where secondary electron effects are most pronounced [170]. Simulations and experimental studies suggest that while larger nanoparticles (~50 nm) achieve a higher DER due to increased gold content, the benefits plateau as self-absorption within the particle reduces additional dose enhancement. For instance, a study with brachytherapy sources showed that AuNPs larger than 50 nm led to a marginally improved DER at the expense of localized tissue overexposure [173]. Some examples of studies on the use of surface-modified AuNPs to improve radiation therapy are reported in Table 3.

Coating involves encapsulating the nanoparticle surface with specific materials, such as polymers (e.g., PEG), surfactants, or organic entities, which provide stability, protection from degradation, and optimize dispersion in various biological environments. Functionalization, on the other hand, involves covalently attaching molecules or functional groups to the nanoparticle surface, giving them specific response capabilities or the ability to bind to defined targets like cells or proteins. This approach also enhances the diagnostic and therapeutic capabilities of the nanoparticles, for instance by attaching antibodies to selectively target cancer cells.

**Figure 6:**
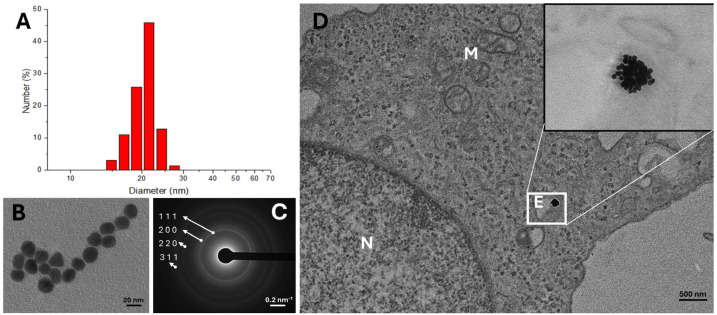
Size distribution (**A**) and TEM micrograph (**B**) of AuNPs and related SAED pattern (**C**). TEM micrograph of U87MG glioblastoma cells incubated with AuNPs for 24 h. AuNPs are highlighted in an endosomal compartment, as indicated in the magnification (**D**). N = nucleus, M = mitochondria, E = endosomal compartment.

In this regard, we are working, within the framework of the Bio Open Lab project, to investigate the potential of immuno/radiation therapy as a treatment for glioblastoma multiforme, a highly aggressive brain tumor. The approach focuses on the use of AuNPs, synthetized by the Turkevich method and functionalized with antibodies. These antibodies are designed to target the nanoparticles specifically to tumor cells, ensuring a more targeted and localized therapeutic effect. By combining these AuNPs with radiation therapy, the goal is to enhance the tumor’s sensitivity to radiation while minimizing damage to surrounding healthy tissue. This targeted strategy holds a promise for improving the effectiveness of glioblastoma treatment and advancing the field of cancer therapy. Preliminary data on AuNPs’ shape, dispersity, and size distribution, evaluated by Dynamic Light Scattering (DLS) (A) and Transmission Electron Microscopy (TEM) (B and C) are reported in Figure 6. The size distribution ranges from 15 to 28 nm and the average size is *d* = 20 nm. Glioblastoma cells (U87MG) are incubated with AuNPs for 24 h to evaluate the internalization. As reported in Figure 6D, AuNPs are engulfed through endocytic pathways. We are currently working on the functionalization of AuNPs with antibodies and the development of the irradiation protocol using protons.

Among the most commonly used coatings is polyethylene glycol (PEG), which improves nanoparticle stability and biocompatibility, extending circulation time in the body, although it may induce immune reactions after repeated administrations [178,179]. Antibodies offer precise specificity for biological targets, but their production can be costly and may require additional modifications to ensure stability and prolonged circulation in the bloodstream. Proteins and peptides can enhance AuNP affinity for specific cellular receptors, though they may be unstable in hostile bodily environments. Amino acids, while more readily accessible, do not always offer the same specificity. Other coatings such as microorganisms (DNA, RNA, aptamers) and carbohydrates present challenges regarding stability and immunogenicity, while polymers offer a wide range of options, although they may face difficulties with biocompatibility.

Finally, gold nanorod synthesis (GNRs) has shown exceptional capability in drug conjugation due to their large surface area, making them reliable platforms for controlled release [180]. However, despite the advantages of these release systems, challenges related to drug stability and release efficiency remain, especially with drugs linked to inorganic nanomaterials.

In the realm of advanced synthesis, methods such as microwave synthesis and gas-phase synthesis have improved control over the size and purity of nanoparticles. Additionally, combining AuNPs with hybrid materials like titanium dioxide and magnetic nanoparticles expands applications in photothermal and magnetic fields, integrating various functionalities into a single platform. Green synthesis continues to gain attention, with the use of complex biomolecules and plant extracts enhancing the biocompatibility and therapeutic activity of AuNPs.

Emerging applications see the use of AuNPs in highly sensitive biosensors for biomarker detection and in smart drug delivery devices capable of targeted drug release in response to internal signals. Future prospects include integrating AuNPs with artificial intelligence (AI) technologies to optimize nanoparticle design and behavior.

Finally, the choice of coating plays a crucial role in ensuring the performance of AuNPs, with each coating presenting advantages and challenges: PEG improves stability and circulation time but may induce immune reactions after repeated administrations; antibodies can be costly and unstable; proteins and peptides might present stability issues; amino acids and carbohydrates may not offer the same specificity; while microorganisms, DNA/RNA, and cellular membranes can have limitations related to stability and immunogenicity.

These developments and innovative approaches represent the latest advances in the field of AuNPs, offering new opportunities and challenges for their application in biomedicine and beyond.

### 7.2. Emerging Techniques in PT with AuNPs

PT represents an advanced form of radiotherapy that employs protons for tumor treatment, offering enhanced precision in dose delivery and reduced exposure of surrounding healthy tissues compared to conventional X-ray radiotherapy. Recent advancements in PT emphasize its potential integration with nanoparticles, particularly AuNPs, to further optimize treatment efficacy and precision.

Protons uniquely concentrate their energy at the Bragg peak, delivering higher doses to malignant tissues while sparing adjacent normal tissues. However, despite tens of thousands of patients having undergone PT, robust clinical evidence fully establishing its superiority over photon-based therapies remains limited [181]. Factors contributing to this uncertainty include the nascent stage of the technology, limited long-term data, and the predominance of passive scattering PT (PSPT), which offers less control than intensity-modulated radiation therapy (IMRT) [182,183].

Intensity-modulated proton therapy (IMPT) represents a significant advancement in radiotherapy, offering enhanced precision by modulating both beam energy and distribution. This precision facilitates superior differentiation between tumor tissues and surrounding healthy structures, paving the way for improved therapeutic outcomes. Research is actively addressing the complexities of PT’s biological effects, focusing on the development of accurate relative biological effectiveness (RBE) models and predictive frameworks tailored to individual patient responses. These innovations are aimed at maximizing treatment efficacy while minimizing the risks of toxicity and recurrence. The integration of AuNPs with advanced therapeutic technologies represents a transformative approach in cancer treatment, leveraging their unique properties to enhance both therapeutic efficacy and diagnostic accuracy. In the field of immunotherapy, AuNPs can be functionalized to modulate the tumor microenvironment, polarizing tumor-associated macrophages toward a pro-inflammatory phenotype, thereby boosting anti-tumor immune responses. For instance, macrophages loaded with dendrimer-entrapped gold nanoparticles have demonstrated enhanced therapeutic outcomes in the combined immunotherapy and chemotherapy of osteosarcoma [184]. In theranostics, AuNPs have been engineered to simultaneously enable advanced diagnostic imaging and localized therapeutic delivery. Multifunctional platforms, such as albumin and gadolinium-coated hollow gold nanoshells, have shown capabilities in quadmodal imaging—including near-infrared fluorescence, photoacoustic imaging, computed tomography (CT), and magnetic resonance imaging (MRI)—while facilitating combined photothermal and photodynamic cancer therapy. This integrated approach significantly enhances treatment monitoring and planning precision [185]. In the domain of multimodal imaging, AuNPs have been employed to improve diagnostic accuracy using modalities such as CT, MRI, and photoacoustic imaging. Magnetic gold nanoparticles, for example, have demonstrated high biocompatibility and tumor-specific targeting, enabling precise tumor localization and guiding near-infrared laser-based therapies [186].These advancements underscore the versatility of AuNPs in bridging imaging and therapeutic innovations, positioning them as critical components in the evolution of personalized oncology and precision cancer treatments.

Furthermore, studies are delving into the immunological differences between proton and photon therapies to devise strategies that counteract immunosuppressive effects. For instance, Mohan et al. provide a comprehensive analysis of IMPT’s precision and its potential for therapeutic refinement through more robust RBE models. Their work highlights how personalized approaches in PT could optimize dose delivery and immunological outcomes, advancing the field toward a more patient-centric paradigm of cancer treatment [187].

Technological innovations in PT also include advanced imaging systems and real-time adaptive techniques. Recent research highlights the implementation of image-guided systems that align therapeutic beams with tumor targets more accurately, compensating for patient movement and anatomical changes during treatment. For instance, Arjomandy et al. discussed the successful incorporation of a novel image-guided system within a PT facility, showcasing enhanced quality assurance measures that align with TG-142 guidelines to improve treatment accuracy and safety [188].

Another pivotal innovation involves the potential for real-time MRI-guided PT, as described by Oborn et al. By integrating MRI systems, clinicians could achieve superior visualization of soft tissues during treatment, enabling adaptive therapy that dynamically adjusts to changes in patient anatomy or tumor position. This technology represents a significant leap forward compared to traditional methods, where imaging limitations have hindered precision in dose delivery [189].

A transformative frontier in PT involves the integration AuNPs, which serve to amplify the therapeutic potential of proton beams. AuNPs possess unique optical and radiological properties that make them ideal radiosensitizers and imaging contrast agents. For instance, Iyer et al. demonstrated how AuNPs can reprogram tumor-associated macrophages when combined with PT, polarizing them toward a pro-inflammatory phenotype. This dual approach enhances both direct cytotoxic effects and immune-mediated anti-tumor responses, addressing a major barrier in resistant tumor types [190]. Moreover, Torrisi et al. demonstrated the use of laser-ablated AuNPs to achieve enhanced imaging and dose accuracy during PT. Their research showed how the unique optical and radiological signatures of AuNPs could be exploited for better treatment planning and real-time dose verification, ensuring that radiation is precisely targeted at tumor sites while sparing healthy tissues [191].

The diagnostic potential of AuNPs further extends to imaging techniques such as computed tomography (CT). Luo et al. demonstrated that AuNPs provide superior contrast compared to traditional agents, selectively accumulate in tumor tissues via the enhanced permeability and retention effect, and improve tumor boundary delineation. These capabilities facilitate precise tumor localization and real-time monitoring, advancing precision in radiotherapy planning [192].

In conclusion, the incorporation of AuNPs into PT represents a significant evolution in radiotherapy, promising to enhance therapeutic outcomes through improved targeting, precision, and integration with complementary treatments. Continued research and clinical validation will be essential to translate these advancements into routine clinical practice, paving the way for innovative and personalized cancer therapies.

## 8. Conclusion

PT represents a significant advancement in the field of cancer radiotherapy, offering unparalleled precision and reduced toxicity compared to conventional photon-based therapies. The unique physical properties of proton beams, particularly the Bragg peak, enable precise tumor targeting while minimizing collateral damage to surrounding healthy tissues. Despite these advantages, challenges persist, particularly in enhancing the biological effectiveness of PT and overcoming technological limitations.

AuNPs emerge as a promising adjunct to PT, demonstrating the potential to amplify therapeutic effects through their radiosensitizing properties. By increasing DNA damage and generating reactive oxygen species (ROS) upon proton irradiation, AuNPs enhance the biological impact of PT. Furthermore, their functionalizability allows for targeted delivery, potentially improving tumor specificity and reducing off-target effects. Preclinical studies underscore the potential of combining AuNPs with PT to delay tumor progression and enhance overall treatment outcomes.

However, translating these findings into clinical practice requires addressing several challenges, including optimizing nanoparticle formulations, ensuring safe and effective delivery, and conducting comprehensive clinical trials. Future research should focus on refining the integration of AuNPs with PT to achieve consistent and reliable outcomes, ultimately expanding the therapeutic applications of this innovative approach.

In conclusion, the combination of PT and AuNPs holds immense potential to revolutionize cancer treatment by offering more precise, effective, and personalized therapeutic options. Continued advancements in this field are essential to realize the full clinical potential of this promising synergy, ultimately improving the quality of life for cancer patients worldwide.

## Figures and Tables

**Table 1:** Advantages and disadvantages of types of metallic nanoparticles used as radiosensitizers.

Nanoparticle Type	Atomic Number (Z)	Key Mechanism	Advantages	Challenges	References
AuNPs	79	Photoelectric effect, Compton scattering	High biocompatibility, dual-function as imaging/radiosensitizer	High synthesis cost	[24]
PtNPs	78	ROS generation, DNA crosslinking	Strong oxidative potential	Cytotoxicity to non-tumor cells	[25]
TiO₂ NPs	22	Photocatalytic ROS generation	Biocompatible, cost-effective	Requires UV or X-ray activation	[26]
GdNPs	64	Secondary electron generation	MRI contrast enhancement	Toxicity without chelation	[27]
AgNPs	47	ROS generation	Strong antimicrobial properties	High cytotoxicity due to ion release	[28]
IONPs	26	Magnetic targeting, ROS generation	MRI contrast enhancement, magnetic targeting potential	Lower atomic number, reduced dose enhancement compared to AuNPs	[29]

**Table 2:** Summary of nanoparticle-assisted radiation therapy findings across cancer models.

Cancer Model	Nanoparticle Size/Coating	Radiation Dose	Findings	References
Epidermoid carcinoma	4 nm/Cetuximab-conjugated AuNPs	2 Gy	Enhanced radiosensitization, increased DNA damage, cell death	[130]
Prostate cancer	27 nm/Goserelin-conjugated AuNPs	5 Gy	Improved tumor targeting, delayed growth (11–32 days), increased survival (36–74%)	[131]
Colon cancer	1.9 nm/Non-coated	up to 4 Gy	Enhanced radiosensitization (SER of 3.78), increased ROS production (234%), and DNA damage in the Bragg peak region	[132]
Glioblastoma	15 nm/LDLR-ligand peptide-conjugated AuNPs	4 Gy	Enhanced therapeutic efficacy by 67–75%, improved tumor targeting, and reduction in TME invasion	[133]

**Table 3:** In vitro studies on functionalized AuNPs to improve radiation therapy.

No.	Surface Modification	Size	Radiation Dose	Cell Line	References
1	PEG	6.1 nm	up to 10 Gy	EMT-6 and CT26 cell	[143]
2	PEG	4.8–46.6 nm	5 Gy	HeLa cells	[70]
3	Citrate or PEG	5 and 5 nm	2, 4, or 6 Gy	PC3, A549, and U2OS cells	[19]
4	Folic acid	15 nm	2, 4 and 6 Gy	LNCAP and HUVEC cells	[174]
5	Niosomes	38.85 and 127.8 nm	X-ray	A549 cells	[175]
6	Iron oxide	5–10 nm	2 Gy	L929, HeLa, and PC3 cells	[176]
7	Gadolinium	5 nm	4 Gy	HeLa cells	[177]
